# Cardiac papillary fibroelastoma arising from the opening of the left inferior pulmonary vein in left atrium: A rare case report

**DOI:** 10.1097/MD.0000000000018281

**Published:** 2019-12-10

**Authors:** Na Lu, Na Wei, Lei Wang, Ye Yuan

**Affiliations:** a The Department of Pediatrics; b The Department of Operation Room; c The Department of Cardiovascular Surgery; d The Department of Anesthesiology, the First hospital of Jilin university, Changchun, China.

**Keywords:** cardiac surgery, cardiac tumor, echocardiography, left atrium, papillary fibroelastoma

## Abstract

**Rationale:**

Cardiac papillary fibroelastoma is a small, benign endocardial tumor, while it is clinically important because of its strategic position and propensity for causing embolic events and hemodynamic complications.

**Patient concerns:**

A 59-year-old female presented our hospital for investigation and treatment of a sudden onset of syncope lasted about 2 minutes.

**Diagnoses:**

Cardiac papillary fibroelastoma arising from left inferior pulmonary vein in left atrium.

**Interventions:**

The tumor was successfully removed by cardiac surgery.

**Outcomes:**

The patient's postoperative course was uneventful, and she was discharged 10 days after surgery. The patient remained free of neurologic deficits and had no evidence of residual or recurrence of tumor with echocardiography during 1 year of follow-up.

**Lessons:**

Cardiac papillary fibroelastoma is a benign tumor, with increased risk of thromboembolic events. It is often diagnosed in patients with echocardiography by chance or after a neurologic event. Complete surgical resection should be considered when the patient is indicated and the long-term postoperative prognosis is excellent.

## 1 Introduction

Cardiac papillary fibroelastoma, the third most frequently occurring benign primary cardiac tumor after myxoma and lipoma, typically affects the cardiac valves, mainly the aortic and mitral valves and very rarely the endocardium of cardiac chambers.^[1]^ Although the tumor is usually small and asymptomatic, exhibits no local infiltration on histopathologic examination, and carries a very low likelihood of recurrence, it may have a malignant propensity for life-threatening complications, mainly neurologic and cardiovascular accidents, such as stroke, myocardial ischemia/infarction, and even sudden death. While all these events are rarely directly responsible for valve dysfunction.^[1]^ The incidence and diagnosis of papillary fibroelastoma is increasing mainly due to the improvement of imaging tools, especially transthoracic echocardiography (TTE)^[2]^ and transesophageal echocardio-graphy (TEE).^[3]^ The only definite treatment with clear benefits is carefully surgical excision of all tumors, usually with excellent results.

The patient reported here presented with a sudden syncope, with no cardiac or other signs or symptoms of general discomfort. Echocardiography revealed a hyperechoic mass originating from the opening of the left inferior pulmonary vein in the left atrium, which was a very rare location of the tumor. The tumor was successfully removed by cardiac surgery, and papillary fibroelastoma was confirmed by histopathologic examination.

## 2 Case report

**Figure 1:**
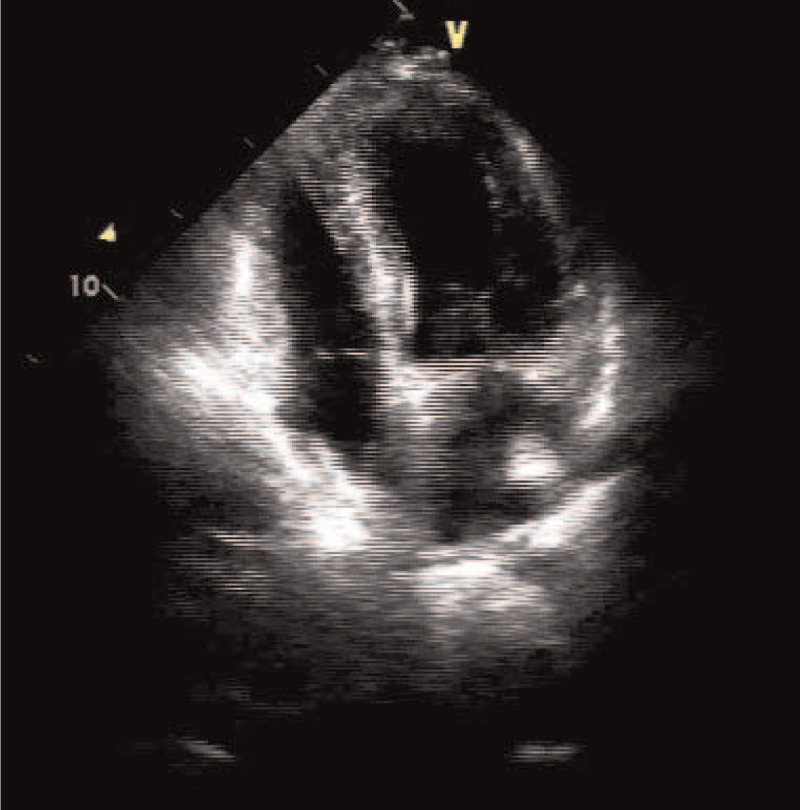
Transthoracic echocardiography demonstrates a 10 × 15 mm mobile hyperechoic mass arising from the opening of the left inferior pulmonary vein in left atrium.

A 59-year-old female presented our hospital for investigation and treatment of a sudden onset of syncope lasted about 2 minutes. She did not have significant past medical history and had taken no medication, and her physical examination was unremarkable. The head computed tomography scan revealed lacunar infarcts, and magnetic resonance imaging showed no evidence of severe stenosis or occlusion in intracranial and carotid arteries. Electrocardiogram showed normal sinus rhythm, and laboratory results were almost within normal limits. Transthoracic echocardiogram revealed a hyperechoic irregular solid structure arising from the opening of the left inferior pulmonary vein and protruding into the left atrium, and the cardiac structure and function were otherwise almost normal (Fig. 1).

**Figure 2:**
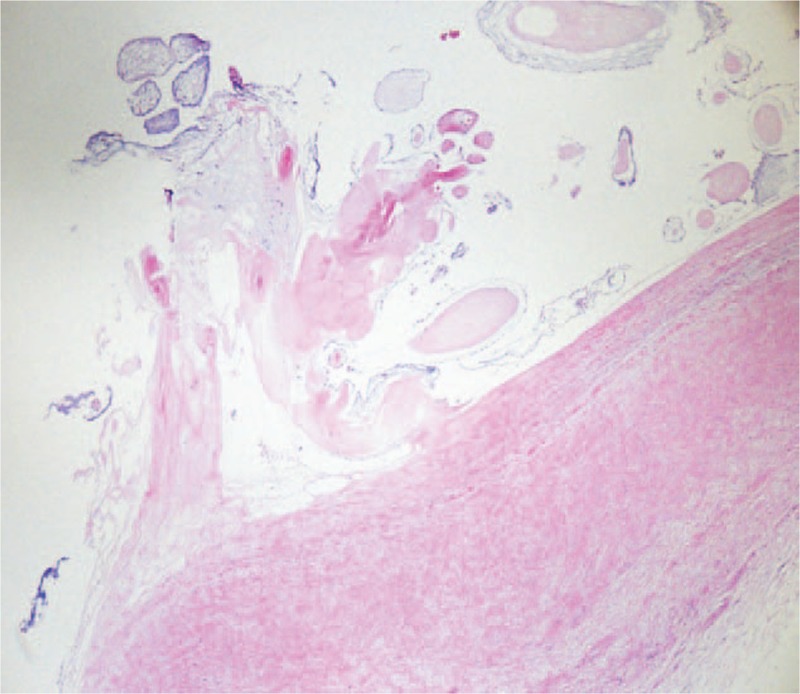
The histological appearance of the papillary fibroelastoma in left atrium.

She was scheduled for elective resection of this mass. Median sternotomy was performed and cardiopulmonary bypass was established via aorto-bicaval cannulation. The left atrium was incised and a 10 × 15 mm mobile, gelatinous mass was found. The mass was resected en bloc along with the stalk and surrounding tissue of the left atrial wall. On gross examination, the tumor was grey-white and soft, and appeared frond-like and translucent when submerged in liquid. Postoperative histopathologic examination showed that the mass was composed of branching avascular papillary fronds lined by a layer of endothelial cells with variable amount myxoid change, collagen, smooth muscle cells and elastic fibers, leading to the definite diagnosis of papillary fibroelastoma (Fig. 2). The patient's postoperative course was uneventful, and she was discharged 10 days after surgery. The patient remained free of neurologic deficits and had no evidence of residual or recurrence of tumor with echocardiography during 1 year of follow-up.

## 3 Discussion

Primary cardiac tumors are uncommon, and the majority of them are benign. Papillary fibroelastoma comprises 4.4% to 8% of these tumors and is the third most frequent benign tumor of the heart, after myxoma and lipoma. The mean age at detection is around 66 years.^[1]^ The risk increases with age, although papillary fibroelastoma can occur in any age groups. The precise etiology remains elusive, and the hypothetical pathogenesis includes the hemo-dynamic stress of blood flow along the endocardium and a possible disposal of fibrin fibers which are used to form organized thrombus. The diagnosis of papillary fibroelastoma is increasing and shifts from an incidental intraoperative or autopsy discovery to ante-mortem detection, mainly due to increasing use of echocardiography, better technology with improved resolution and enhanced awareness of the tumor. Moreover, cardiac magnetic resonance imaging may contribute to better diagnosis and differentiation of the tumor from other cardiac masses.^[4]^ A recent large-scale study about bioepidemiologic implications of papillary fibroelastoma found that papillary fibroelastoma was more common than cardiac myxoma, at a rate of approximately 2:1, making it the most common benign primary cardiac neoplasm of adulthood.^[1]^

Papillary fibroelastoma typically originates from the valvular endocardium (60%–84%) on the flow surface, and is the most common valvular neoplasm.^[5]^ The aortic valve is the most often affected site (59%) followed by the mitral valve (13%), the tricuspid valve (4%), and the pulmonary valve (2%).^[1]^ The largest retrospective analysis of 725 patients revealed the valvular surfaces as the most common location of the tumor, with only 10 arising from the left atrial wall.^[6]^

Although histologically benign and clinically asymptomatic, papillary fibroelastoma may cause devastating thromboembolism-associated complications, such as stroke, angina, myocardial infarction, syncope, and peripheral infarcts including splenic, retinal, mesenteric, and even sudden death.^[6–9]^ The clinical presentation is determined by many factors, mainly the tumor location, size, growth rate, and tendency for embolization. The incidence of complications has been reported as high as 53% and is higher than other benign cardiac tumors such as myxoma, lipoma,^[10]^ or even vegetation.^[11]^ The embolic phenomena are presumably secondary to formation of fibrin thrombi along the tumor papillae and its dislodgement.

Echocardiography is a convenient and non-invasive diagnostic technique and should be the first choice of tests to search for papillary fibroelastoma.^[12]^ Diagnosis is usually made by TTE and TEE, with images confirming a pedunculated and mobile mass attached to the valve or endocardium, with refractile appearance and stipple edge.^[2,3]^ The computed tomography is inferior to the echocardiography in depicting the small moving structures. Cardiac magnetic resonance imaging is a helpful imaging modality that can identify important aspects of intracardiac tumors, including location, size, tissue characterization, and enhancement patterns.^[13,14]^. Histologic examination shows avascular structures consisting of elastic fibers, fibrous tissue, and collagen fibers with an outer layer of regular endothelial cells.^[15]^

There are no clear guidelines for papillary fibroelastoma treatment, although the only definite treatment with clear benefits is completely surgical excision of all tumors. Decision of surgery depends on the size, location, mobility, and the association of the tumor with symptoms, as in our case. Surgical intervention should be recommended if papillary fibroelastoma is symptomatic, mobile, or in large size (>10 mm).^[8]^ If the patient is not a surgical candidate or refuses surgery, or the patient is awaiting for surgery, anticoagulation with warfarin or antiplatelet therapy with aspirin should be administered to prevent thrombo-embolism. Asymptomatic patient with nonmobile papillary fibroelastoma should be followed-up closely. The completely surgical resection of papillary fibroelastoma with preservation of native valve function has a high success rate and is classified as a safe and well-tolerated procedure.^[16–19]^ After excision, recurrence is very rare (1.6%).^[1]^ The postoperative courses are usually uneventful, and short- and long-term outcomes after surgery are generally excellent.

## 4 Conclusion

In conclusion, papillary fibroelastoma we presented arising from the opening of the left inferior pulmonary vein in left atrium is a very rare benign cardiac tumor, with increased risk of thromboembolic events. It is often diagnosed in patients with echocardiography by chance or after a neurologic event. Completely surgical resection should be considered when the patient is indicated and the long-term postoperative prognosis is excellent. Further clinical trials are needed to understand the mechanisms of tumor pathology, to define the proper patients for surgery, and to determine whether medical treatment with antiplatelet or anticoagulation will improve outcomes.

## Author contributions

**Conceptualization:** Na Lu, Na Wei, Ye Yuan.

**Data curation:** Na Lu, Na Wei, Lei Wang, Ye Yuan.

**Formal analysis:** Na Lu, Ye Yuan.

**Investigation:** Na Lu.

**Methodology:** Na Lu, Na Wei, Lei Wang.

**Resources:** Na Lu, Na Wei.

**Software:** Na Wei, Lei Wang.

**Supervision:** Na Lu, Ye Yuan.

**Validation:** Na Lu, Lei Wang, Ye Yuan.

**Visualization:** Na Lu, Na Wei, Lei Wang, Ye Yuan.

**Writing – original draft:** Na Lu, Na Wei, Lei Wang, Ye Yuan.

**Writing – review & editing:** Na Lu, Na Wei, Lei Wang, Ye Yuan.

Ye Yuan orcid: 0000-0002-5015-5719.
